# How the geometry of the scan body affects the accuracy of digital impressions in implant supported prosthesis. *In vitro* study

**DOI:** 10.4317/jced.59948

**Published:** 2022-12-01

**Authors:** Carlos Alvarez, Pablo Domínguez, Emilio Jiménez-Castellanos, Gema Arroyo, Ana Orozco

**Affiliations:** 1 DDS, PhD Student, Department of Prosthodontics, College of Dentistry, University of Seville, Seville, Spain; 2 DDS, PhD. Clinical Associate Professor, Department of Prosthodontics, College of Dentistry, University of Seville, Seville, Spain; 3 DDS, PhD. Professor and Head, Department of Prosthodontics, College of Dentistry, University of Seville, Seville, Spain; 4 DDS, PhD. Clinical Associate Professor, Department of Prosthodontics, College of Dentistry, University of Seville, Seville, Spain

## Abstract

**Background:**

To determine and compare how three-dimensionally accurate scan bodies of different geometric shapes are placed over 6 implants (platform or crestal module).

**Material and Methods:**

A master plaster model was made with 6 INHEX STD implant analogs made by Mozo-Grau S.A and 4 scan body types were compared. Several groups were made: a control group using a DS101 85G20 contact scanner (Renishaw, Gavá, Spain) and 2 experimental groups using optical scanners: Cerec Omnicam (Sirona, Bensheim, Germany) and Trios 3 (3Shape, Copenhagen, Denmark). 3 parameters were measured on the implants: dis-tance between the axial axes, height difference and angulation difference. Two experienced op-erators scanned 10 times using each of the 2 scanners. The STL files were compared using the “Best-Fit” technique and the data was then extrapolated and processed statistically.

**Results:**

The scan bodies PRMG (SB3) and TALL (SB4) lead to smaller errors in distance, projected height and angulation than ELOS (SB1) and MG (SB2).

**Conclusions:**

Despite the results obtained in PRMG (SB3) and TALL (SB4), the scanning errors may still be too large to achieve a good fit in large rehabilitations over implants. Any marginal discrepancy may lead to the failure of the rehabilitation or the implant due to the associated biomechanical problems.

** Key words:**IOS, CAD/CAM, SCAN Bodies.

## Introduction

Scan bodies are precision attachments that are generally screwed to the coronal part of the implant to reproduce its position in the digital model that is produced with an intraoral scanner (1).

The literature on how the scan body influences the digital scanning process (2-5) is insufficient. Other factors not linked to the scan body may also condition it, such as temperature, humidity or ambient pressure (recommended: temperature 20º ±1, humidity 55% ±3, and pressure 761 ± 5 mmHg.) (6); these may affect parameters like accuracy and precision and/or resolution. Other contributing factors are: ambience light (7,8), operator skill or the type of scanner used (9,10).

In terms of how the scan body affects the result, there is some evidence that its geometry may be a determining factor, since polished surfaces are easier to scan than irregular or corrugated ones. It has also been found that when the change of surface is more abrupt, such as in very marked edges, the errors recorded are greater (11).

The material the scan bodies are made of may also be a factor, but the only existing evidence found is that the data obtained by an IOS is more accurate the more opaque the scanned material is (12), and the scanning results seem faulty when carried out on metallic surfaces (13).

Parameters such as the angulation between scan bodies may interfere with accuracy and precision (14), as well as the design or engineering tolerance on its fabrication (15,16). The tolerance range that scan bodies impressions have can also affect the result. Recent studies show lateral variations of up to 0.25mm in some scan bodies, which may affect the fit of the final prosthesis and eventually lead to biomechanical issues such as mucositis or peri-implantitis (17,18).

The aim of this study is to determine and compare the accuracy of the scan bodies with different geometric shapes in a model with 6 implants. Parameters measured for this purpose: distance between the center of the working planes of the 6 implants, angle between the insertion axes of the 6 implants and projected height (The projected height of the center of the working plane of an implant “B” on the reference frame of the working plane of an implant “A” is calculated by means of the scalar product of the vector position of the implant “B” in the reference frame “A” on the vertical direction vector of the reference frame of the working plane of the implant “A”).

## Material and Methods

An experimental *in vitro* study was designed, where the independent variable was the type of scan body.

**Figure 1:**
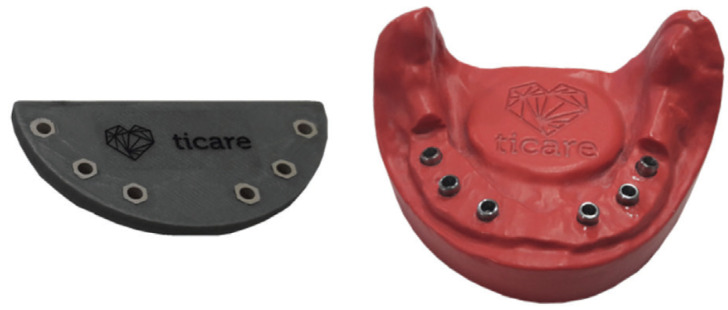
Implant placement guide and polyamide model.

A polyamide master plaster model was created using the HP JET FUSION printer (HP Inc., Palo Alto California, USA) with the space for the implant analogs INHEX STD (ref. 23205501) made by Mozo Grau S.A., in the position of 36, 34, 32, 42, 44 and 46. A polyam-ide splint was created using a 3D printer to attach the analogs to the plaster model with cyanoacrylate (Fig. 1).

The distance between 36, 34, 32 and 46, 44, 42 was 10mm and the angle 0º. The distance between 32 and 42 was 20mm and the angle 15º, which could be considered a borderline case of non-corrected angulation.

A first scanning of the model was registered to determine the control group using a Renishaw DS101 85G20 Contact Scanner (Renishaw DS10), a coordinate measuring machine (CMM) which had already been used in previous studies whose contact probe has a diameter of 1mm and an accuracy of 20um (16). The fabricated model and the scanbodies were scanned five times until the position and the direction of the vector were exact and the uncertainty level was considered adequate using a touch-trigger probe (Contact Scanner - Renishaw DS10).

To carry out this study, 2 intraoral scanners were used: Cerec Omnicam (Sirona, Bensheim, Germany) and Trios 3 (3Shape, Copenhagen; Denmark). Complex and symmetry geometrically shaped scan bodies were used, as well as simple and asymmetric.

**Figure 2:**
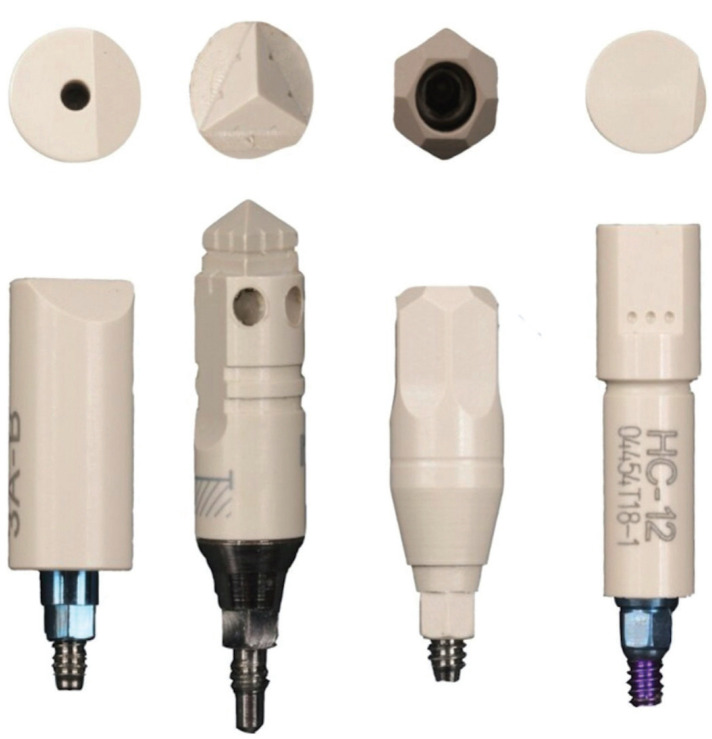
Scanbodies ELOS, MG, Ticare MG and Talladium.

The scan bodies used were made of polyether ether ketone (PEEK), an opaque white material, and had an interior space for a titanium screw to fixate it to the analog/implant with a 5N/cm2 torque with a dynamometric key. Several scan bodies were used for the purpose of this study. The model 3a-B ELOS Medtech Denmark (ELOS) comes in one piece which is screwed in and has a milled angulated side. Two Mozo Grau S.A scan bodies were used: one with a milled pyramidal side, screw-in placement, and two-piece clip in system (MG) and the other one with 12 milled sides, screw-in placement and one piece (Ticare MG). Finally, the Talladium scan body (Talladium Spain) had a milled side, magnetic placement and 2 pieces (Fig. 2).

The working method was always the same.

1. Scanning the model 10 times, by the 2 operators and using the different scanners (experimental groups). A one-step scanning technique was used (19).

2. Exporting of the STL file from the scanner to the dental design software Exocad (DentalDB 1.0 5585).

3. Scan processing and “best fit” with the existing library.

4. Exporting the plaintext file from the dental processing software to the statistical processing software using the Student´s T-test for paired samples to compare the data obtained by the 2 operators and the 2 scanners, and the ANOVA test to compare the scan bodies. If significant differences were found, the Student´s T-test for paired samples with Bonferroni correction was carried out (following an assessment of the equality of variances with Levene’s test).

## Results

Comparison between the errors made by both operators.

Significant differences were found between both operators only when considering the errors in angle (*P*<.01). The first operator was more accurate, with an average of 0.107 ± 0.330 degrees (CI 95%).

Comparison between the errors made by both scanners.

Significant differences were found between both scanners for the three studied parameters (*P*<.01), with the Trios 3 being the most accurate, with a mean error of 0.019 ± 0.185 (CI 95%) mm in distance, 0.377 ± 0.093 (CI 95%) in angulation, and 0.043 ± 0.012 mm (CI 95%) in height.

Comparison between the errors made when comparing the scan bodies.

 Table 1,  Table 2 and  Table 3 show the mean differences and the standard deviation of the errors in distance, angle and height between the four Scan Bodies. It can be seen that the Ticare MG Scan Bodies and the Talladium are more accurate for the three parameters than the other two (ELOS and MG).

**Table 1:**
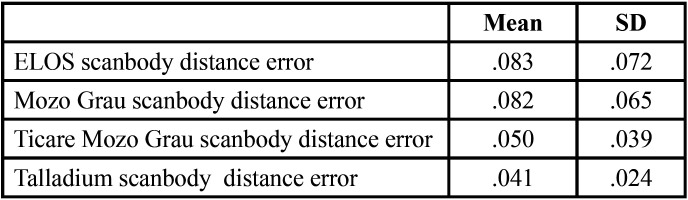
Mean and standard deviation of the distance errors in mm between the 4 scan bodies.

**Table 2:**
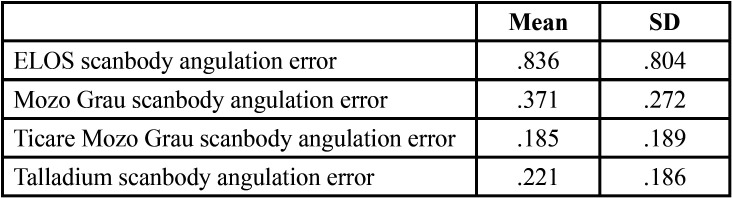
Mean and standard deviation in the errors in angulation between the 4 scanbodies.

**Table 3:**
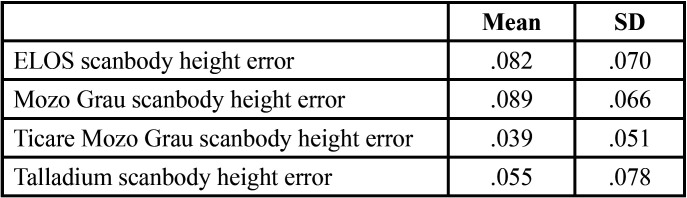
Mean and standard deviation in mm of height errors between the 4 scan bodies.

The main average differences found were.

- In distance, between ELOS and Talladium, with Talladium providing better results (0.041 ± 0.013 mm CI 95%).

- In angulation: between ELOS and TicareMG, with the latter being more accurate (0.644 ± 0.143 degrees IC 95%).

- In height: between MG and TicareMG, with the second one being more accurate (0.051 ± 0.015 mm IC 95%).

When doing the distance inferential analysis, significant differences were found of ELOS with Ticare MG and Talladium (*P*<.01). In angulation errors, significant differences were found between ELOS and MG, and also between TicareMG and Talladium (*P*<.01). In terms of projected height, significant differences were found between ELOS and MG, and also between TicareMG y Talladium (*P*<.01).

**Figure 3:**
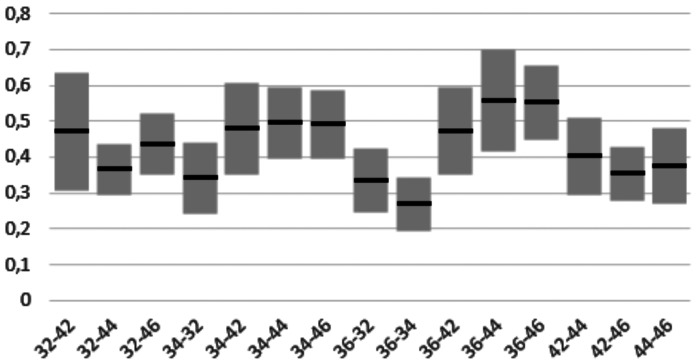
Distance errors in mm between the height of the different implants.

**Figure 4:**
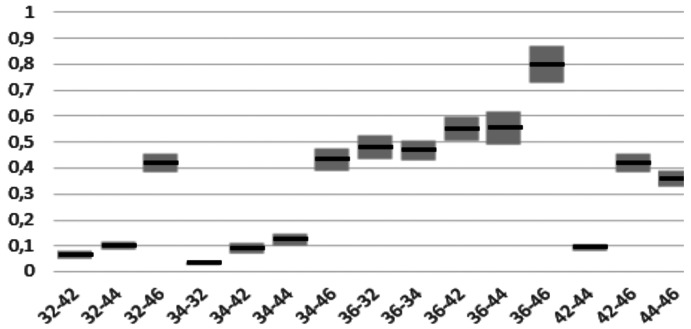
Angulation errors in degrees between the height of the different implants.

**Figure 5:**
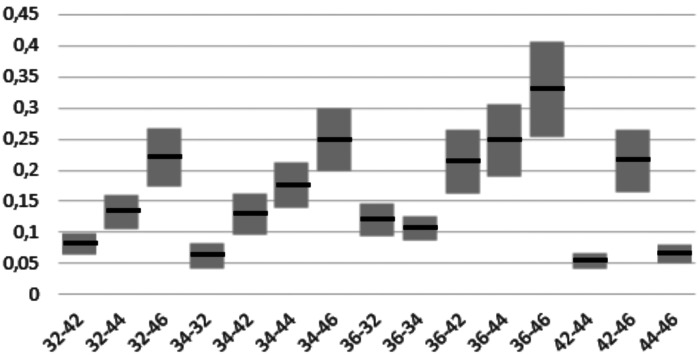
Height errors in mm between the height of the different implants.

Finally, the errors found in the three studied parameters between the positioning of the 6 implants can be seen in Figs. 3-5. This shows that the errors seem to increase the further the implants are one from another, especially in distance and height.

Distance: average error between 36- 46 of 0.798; 36-44 of 0.555; and 36-42 of 0. 551 mm. Angulation: average error between 36-44 of 0.558; 36-46 of 0.552; and 34-46 of 0.490 degrees. Height: average error between 36-46 of 0.329 mm; 34-46 of 0.248; and 36-44 of 0.248 mm.

## Discussion

-Methodology discussion.

This study was carried out “*in vitro*” to eliminate factors that could influence the intraoral scanning and affect the obtained measurements, but it obviously has some limitations. In future studies, it would be interesting to do an “*in vivo*” study in non-ideal conditions (5-7), and where the influence of soft and mobile tissues was taken into account although we should point out the complexity of these conditions given the impossibility of using a CMM in the oral environment.

One of the factors that may have caused discrepancies in the measurements are the variations and tolerances when making the scan bodies. In this study, each scan body was placed on its analog in the model as recommended by the maker. However, the contact scanner was not used to measure after each scanning, which could cause inaccuracies, because screwing and unscrewing can cause deformations after more than just 10 uses (8).

Another factor could be the use of analogs. Stimmelmayr *et al*. (1) analyzed “*in vitro*” the accuracy of the measurements registered with a lab scanner of the same type of scan body and on the same model with 4 implant analogs and 4 implants. The results showed a discrepancy of 39 ±58 μm in the original implants and 11±17μm in the analogs, which could lead to thinking that scanning with the implant analogs could also influence the result. In the above said study the scan bodies were screwed in with a dynamometric key, which has been shown to be less precise in their placement (8).

The scan bodies selected were as different from each other as possible, since it could influence the accuracy of the measurement, as thought by Mizumoto *et al*. (2) Fluegge *et al*. (14) determined on their study that the wider and longer the scanned surface was, the more accurate the scanning would be, and therefore the determination of the correct position of the implant.

Our scan bodies are made out of PEEK, since intraoral scanners do not register well information on metallic and reflective surfaces against opaque ones, causing numerous “holes” (10,12). This material has a neutral color with a high value (it is light in color) which is similar to improved stone plaster, and an adequate shine on surface to be measured using optical scanners (19). It is very stable three-dimensionally, and suffers very little modifications with temperature changes. It is also easy to mill, which made it easy to make model 3 of the scan body (PR1039, Ticare Mozo Grau S.A. Spain). This scan body was a prototype designed based on previous studies that support that the more sides an object has the better interpolated it is (9), since the scanners measure individual points and tend to generate more discrepancies in areas with angles (11) damaging the results.

Therefore, a structure was designed with multiple large surfaces to allow better repositioning in the virtual model and removing the inaccuracies that areas with angles may generate.

The scan bodies were screwed in with a 5N/cm2 torque with a dynamometric key. This tool does not allow an exact precision, which may cause discrepancies. It could even decalibrate itself through use, causing even more discrepancies.

The contact scanner Renishaw was chosen as the tool to obtain the reference values since it is considered the most precise to verify measurements in solid 3D objects (11).

The radius of the ruby touch-trigger probe of the contact scanner (0.5 mm) was enough to allow stable positioning over the reference points of the real model without causing relevant inaccuracies on these measurements and gave the reference values or “gold standard” of this study. A one step scanning technique was chosen since, according to the literature, it is more accurate than the double scanning technique (19).

Some studies overlap all the STL files and the data groups and calculate the standard deviations (1). On this study, the data obtained from the STL files was compared to the data obtained with the contact scanner using Software Exocad (DentalDB 1.0 5585), likewise Revilla-León *et al*. (3).

There are others methods, like Geomagic Qualify ®software, that could obtain a “best fit”. It is a lineal alignment, which allows a more precise observation of which points of the scanned surface are less accurate and therefore have cause greater maladjustment.

The potential disadvantage that a “best-fit” has is that, focusing in the relation between both files STL with less misfit, a discrepancy in a specific area may be camouflaged and distributed evenly over the rest of the virtual model. This could hide a systematic measuring error in specific areas.

-Discussion of the Results.

Despite not being included in of the goals of the study, two different operators and two scanners were used, to reject the possibility of these affecting the results. Despite statistically significant differences in angulation errors, no clinically significant differences were found depending on the operator, but there were differences depending on the scanner used, as other authors have described (9,10).

About the geometry of the scan bodies:

The surface geometry and dimension requirements of the scan body for a precise transfer of the position of the implant to the virtual model have not been thoroughly studied yet. The existing literature does not provide information regarding the precision in the capture of the scan bodies depending on the different geometry and dimensions of the scan bodies surface. A 2021 literature review about digital impressions in implantology only found 5 articles that link the design of the scan body with the precision of the digital impression (15).

The current study found the worst results using the MG scan body, which has a very complex anatomy with a milled pyramidal side and 2 pieces. This agrees with the publication of Kurk (11). which shows that the more pronounced the surface change is, like in sharp edges, the bigger the errors registered are. Furthermore, the difficulty to scan the whole anatomy of the scan body completely makes the scanning software use algorithms to fill the holes that it has not been able to scan. The image created will have defects or artifacts as described by authors (14), which would negatively affect the best-fit procedure.

The results with the other 3 scan bodies were better since, as indicated by the study of Motel (17) scan bodies with a flatter and simpler structure are linked with significantly smaller devia-tions in the digital impressions. The tolerance margins in their fabrication, such as proven by the study of Lener in 2021, where the greater tolerance deviations were found in scan bodies with conical internal connection (11).

The average discrepancy was the main study variable. It represents how much the position of each point on the STL file could deviate from the data obtained with the Renishaw contact scanner. This could be a good indicator of the accuracy of the scanner, but it does not involve the entire scanned surface and does not provide information of the scanners performance depending on the area of the scan body.

Throughout this study, after data of each of the scanned implants was obtained and the accuracy was evaluated, it was found that the greater the scanning area the greater the inaccuracy.

A strong dependency has been found between the positioning error in distance with the relative position of the implants, with greater errors happening the greater the distance and angulation between the scanned implants. As seen before, the inaccuracy of the digital impressions may be reduced the more scan bodies (implants) on the arch. Also, the difficulty would increase if scan bodies were identical, both for the intraoral scanner to identify their correct posi-tion and for the technique required (13). Mizumoto *et al*. (4) assessed different scanners and scan bodies in an edentulous arch which was rehabilitated with 4 implants, and, even though the implants were fewer, the standard deviation values found were greater than 0.17mm in distance and 0.5º in angulation. This could lead to thinking, that, despite the scanner not having a clinical use to rehabilitate over implants, it could indeed be used for restoring smaller sections, and it would be interesting to study what the length limit would be to obtain a good fit in a partial prosthesis over implants.

Future studies of the prototype PRMG (SB3) would also be interesting, using only one experienced observer and in “*in vivo*” circumstances.

## Conclusions

1. Scan bodies PRMG (SB3) y TALL (SB4) lead to smaller errors in distance, projected height and angulation than the ELOS (SB1) and MG (SB2), with a significant difference.

2. Regardless of the studied parameters, it has been noted that the errors are too large to achieve a good fit in large structures with unparallel implants. Intraoral scanner cameras using the current technique do not provide sufficient precision to ensure a good fit in this type of treatment.

## References

[1] Stimmelmayr M, Güth JF, Erdelt K, Edelhoff D, Beuer F (2012). Digital evaluation of the reproducibility of implant scanbody fitan in vitro study. Clin Oral Invest.

[2] Mizumoto RM, Yilmaz B (2018). Intraoral scan bodies in implant dentistry: A systematic review. J Prosthet Dent.

[3] Revilla-León M, Fogarty R, Barrington JJ, Zandinejad A, Özcan M (2020). Influence of scan body design and digital implant analogs on implant replica position in additively manufacured plaster models. J Prosthet Dent.

[4] Mizumoto RM, Yilmaz B, McGlumphy EA Jr, Seidt J, Johnston WM (2020). Accuracy of different digital scanning techniques and scan bodies for complete-arch implant-supported prostheses. J Prosthet Dent.

[5] Revilla-León M, Jiang P, Sadeghpour M, Piedra-Cascón W, Zandinejad A, Özcan M (2020). Intraoral digital scans-Part 1: Influence of ambient scanning light conditions on the accuracy (trueness and precision) of different intraoral scanners. J Prosthet Dent.

[6] Revilla-León M, Jiang P, Sadeghpour M, Piedra-Cascón W, Zandinejad A, Özcan M (2020). Intraoral digital scans: Part 2-influence of ambient scanning light conditions on the mesh quality of different intraoral scanners. J Prosthet Dent.

[7] Arakida T, Kanazawa M, Iwaki M, Suzuki T, Minakuchi S (2018). Evaluating the influence of ambient light on scanning trueness, precision, and time of intra oral scanner. J Prosthodont Res.

[8] Abduo J, Elseyoufi M (2018). Accuracy of intraoral scanners: a systematic review of influencing factors. Eur J Prosthodont Restor Dent.

[9] Kim J, Park JM, Kim M, Heo SJ, Shin IH, Kim M (2016). Comparison of experience curves be-tween two 3-dimensional intraoral scanners. J Prosthet Dent.

[10] Schmidt A, Billig JW, Schlenz MA, Rehmann P, Wöstmann B (2019). Influence of the Accuracy of Intraoral Scanbodies on Implant Position: Differences in Manufacturing Tolerances. Int J Prosthodont.

[11] Kurz M, Attin T, Mehl A (2015). Influence of material surface on the scanning error of a powder-free 3D measuring system. Clinical Oral Investigations.

[12] Henkel GL (2007). A comparison of fixed prostheses generated from conventional vs digitally scanned dental impressions. Compendium Continuing Education in Dentistry.

[13] Chia VA, Esguerra RJ, Teoh KH, Teo JW, Wong KM, Tan KB (2017). In vitro three-dimensional accuracy of digital implant impressions: the effect of implant angulation. Int J Oral Maxillofac Implants.

[14] Fluegge T, Att W, Metzger M, Nelson K (2017). A novel method to evaluate precision of optical implant impressions with commercial scan bodies. An experimental approach. J Prosthodont.

[15] Marques S, Ribeiro P, Falcão C, Lemos BF, Ríos-Carrasco B, Ríos-Santos JV (2021). Digital Impressions in Implant Dentistry: A Literature Review. Int J Environ Res Public Health.

[16] Lerner H, Nagy K, Luongo F, Luongo G, Admakin O, Mangano FG (2021). Tolerances in the production of six different implant scanbodies: a comparative study. Int J Prosthodont.

[17] Patzelt SB, Emmanouilidi A, Stampf S, Strub JR, Att W (2014). Accuracy of full-arch scans using intraoral scanners. Clin Oral Investig.

[18] Pesce P, Pera F, Setti P, Menini M (2018). Precision and accuracy of a digital impression scanner in full-arch implant rehabilitation. Int J Prosthodont.

[19] Motel C, Kirchner E, Adler W, Wichmann M, Matta RE (2020). Impact of Different Scan Bodies and Scan Strategies on the Accuracy of Digital Implant Impressions Asessed with an Intraoral Scanner: An In Vitro Study. J Prosthodont.

